# Nestling Growth Strategies of Two Sympatric Rosefinch Species in a Tibetan Alpine Habitat

**DOI:** 10.3390/ani16050761

**Published:** 2026-03-01

**Authors:** Yihua Tan, Xin Lu

**Affiliations:** 1 Department of Ecology, College of Life Sciences, Wuhan University, Wuhan 430072, China; tanyihua_sp@126.com; 2 Department of Ecology, College of Life Sciences, Henan Normal University, Xinxiang 453007, China

**Keywords:** alpine environment, *Carpodacus*, life history, growth parameter, Tibet

## Abstract

How ecological factors, particularly food availability and breeding season length, shape growth strategies in ways that can override allometric constraints is a key question in life-history evolution. Here we address this question by comparing body-mass growth of two *Carpodacus* species of distinct body size, the streaked rosefinch *C. rubicilloides* (~40 g) and the pink-rumped rosefinch *C. eos* (~20 g), which commonly nest in the alpine zones of south Tibet. Although nestlings of both species fledged at the same age and at a similar body size relative to the adults, the larger species grew significantly faster than the smaller one, despite an allometric scaling constraint that predicts the opposite. This counterintuitive pattern may be explained by interspecific differences in (i) nestling food availability and (ii) time constraints on growth. Nestlings of *C. rubicilloides* fed exclusively on the seeds of a highly productive leguminous plant, whereas those of *C. eos* consumed a diverse array of small seeds that were expected to require greater searching effort, potentially limiting energy intake. Concurrently, *C. rubicilloides* experienced a shorter breeding window (~30 days) compared to its congener (~50 days), a difference likely linked to species-specific availability of food resources. These observations suggest that, for closely related species coexisting in harsh alpine environments, the effect of ecological factors on growth can override that of allometric constraints in shaping growth trajectories.

## Simple Summary

The present research compared the growth trajectories of two sympatric, size-distinct *Carpodacus* species, namely the larger streaked rosefinch (*C. rubicilloides*) and the smaller pink-rumped rosefinch (*C. eos*), aiming to test the prediction that ecological factors can help organisms to overcome the allometric constraints of body size on growth rate. Logistic growth models showed that, although nestlings of the two species fledged at a similar age, the larger species grew significantly faster than the smaller species, a pattern opposite to what would be predicted from body size. This pattern likely stems from a segregation in nutritional niche, whereby the larger species relied mainly on energy-rich legume seeds, whereas the smaller species consumed smaller, more diverse grass seeds that require greater searching effort. In addition, a stronger seasonal time constraint experienced by the larger species may further accentuate this growth divergence. The result supports the initial prediction and contributes to our understanding of avian life-history divergence in alpine environments.

## 1. Introduction

Of 20 *Carpodacus* species adapted to habitats above the treeline in Asia, the streaked rosefinch *C. rubicilloides* and the pink-rumped rosefinch *C. eos* commonly nest in alpine zones above 4300 m in south Tibet, making them among the world’s highest-altitude passerines [1]. Weighing approximately 40 g and 20 g, respectively, *C. rubicilloides* and *C. eos* represent the largest and smallest members of their genus, respectively [2]. Our previous studies showed that in these alpine zones, both of the size-distinct congeners are present throughout the year, whereas other *Carpodacus* species of intermediate size occur there only during the non-breeding periods, likely a result of avoiding interspecific competition for reproduction-related resources in the extreme environments [2].

Growth trajectories of species are subject to natural selection. Many environmental factors can shape growth strategies, including food availability [3], seasonal timing [4], and ambient temperature [5,6]. These factors act by regulating metabolic processes involved in energetically demanding growth [7]. For sympatric species, food availability stands out as a critical determinant. Like most alpine seed-eating birds in Eurasia [8], both rosefinch species begin breeding approximately two months later than local insectivorous passerines in response to seed ripeness [9,10,11,12]. Trophic niche segregation is considered to be an important mechanism enabling the two sympatric congeners to coexist stably. Specifically, *C. rubicilloides* feeds exclusively on large seeds of a leguminous plant, relying on its strong beak, while *C. eos* consumes small seeds (averaging 1/15 the size of leguminous seeds) of grasses, corresponding to its small beak [13]. It has been suggested that *C. rubicilloides* faces less severe food limitation compared to *C. eos*, because the leguminous species is dominant in local plant communities, and its seeds are highly productive and easy for the birds to access; however, as shown in other granivorous finches [14,15], small seeds would incur higher searching costs on *C. eos* parents, especially in the alpine zones where grasses are less developed (mostly found in streamside habitats or at the base of shrubs) due to dry soil associated with strong solar radiation. Given that food supply is one of the most important environmental causes of variation in nestling growth [16], this interspecific difference in food availability could be primarily responsible for the divergence in growth traits between the two congeneric species.

Seasonal time constraints are another critical driver of growth strategies, with faster growth rates and shorter growth durations being favored when the time window for completing development is narrow [7]. Typically, *C. rubicilloides* exhibits an earlier onset of reproduction and experiences a shorter nesting period compared to *C. eos* (early July vs. mid-July; 14.0 vs. 13.5 days), in response to species-specific availability of food resources [13]. To deal with the tighter time schedule, we expect that, all else being equal, nestling *C. rubicilloides* should grow faster than its congeners to reduce the time spent at the nest.

Body size affects the growth process, because the rate at which biomass can be produced is inevitably slower in larger organisms than in smaller ones. All else being equal, nestlings of larger species exhibit a lower growth rate and experience a longer nestling period than those of smaller species [17,18]. To cope with the tighter seasonal time schedule, *C. rubicilloides* is expected to break the allometric constraint by taking advantage of plentiful food resources to accelerate growth, compared to *C. eos*, which potentially suffers from food limitation but benefits from a wider developmental time window and a relatively weak allometric constraint. In other words, the potentially lower growth rate and longer growth duration of larger-bodied *C. rubicilloides* relative to smaller-bodied *C. eos* will be compensated for by interspecific divergence in trophic niches.

Here we compared nestling growth parameters between the two species to test the prediction that after accounting for the influence of body size, *C. rubicilloides* exhibits a higher growth rate and a shorter nestling period than *C. eos*. In addition, to place the growth patterns of the alpine rosefinches in a broader comparative context, we compared the growth parameters of the two species with body-mass-based passerine allometric predictions and assessed how they differ from published estimates of two closely related lowland congeners.

## 2. Materials and Methods

### 2.1. Study Area and Study Species

This study was conducted in the Xiongse valley (29°27′ N, 91°40′ E, altitude range from 3900 to 5600 m) near Lhasa, Tibet, from July to September of 2002–2004, during the breeding seasons of the two species. The annual average air temperature in the study area is 4.5 °C and annual total precipitation is 566 mm, 80% of which falls between June and September. Vegetation is characterized by alpine shrublands (see [19] for further information).

The two rosefinch species are present in the alpine habitats year-round. They are socially monogamous and non-territorial. Incubation is performed by females only and nestlings are provisioned by both sexes. Males provide food for their mates when the latter are engaged in incubation and brooding of the young. Both species nest only in the rose–barberry vegetation zone that extends from 4000 to 4550 m on the south-facing slopes. More than 90% of nests of both species are built in three common shrub species: the rose *Rosa sericea*, the barberry *Berberis hemsleyana*, and the peashrub *Caragana bicolor*. However, *C. rubicilloides* prefers taller individual bushes whereas *C. eos* more often chooses lower, denser ones. The two species are statistically similar in the following reproductive parameters: annual breeding attempt (1), mean clutch size (*C. rubicilloides*: 3.69 ± 0.66, *n* = 75; *C. eos*: 3.81 ± 0.57, *n* = 108), brood size at hatching (3.30 ± 0.98; *n* = 54; 3.22 ± 0.95, *n* = 76), brood size at fledging (2.85 ± 1.10, *n* = 34; 2.61 ± 1.05, *n* = 41), incubation period (15.3 ± 0.9 d, *n* = 12; 15.0 ± 1.4 d, *n* = 17), and the proportion of nests from which at least one nestling fledged (47%, *n* = 52; 42%, *n* = 93) [13]. When comparing nestling growth in the context of food availability, these similarities allowed us to exclude several potential confounding factors that affect nestling growth, including sibling competition and nest predation.

### 2.2. Field Work

We located rosefinch nests by systematically inspecting potential nesting bushes or by following individuals displaying breeding activity. For each nest found before hatching, we monitored these nests daily, especially around the estimated hatching date, until hatching occurred. Eggs within most clutches of both species hatched within 24 h. Nestlings in a brood were individually marked by clipping specific tufts of down before they reached 8–10 days of age and thereafter with colored leg rings.

Growth of each nestling was monitored daily or every other day from hatching until fledging. We weighed the body mass of each nestling using an electronic scale with an accuracy of 0.1 g and measuring morphological dimensions (bill, wing, tarsus, and tail) using a vernier caliper to 0.1 mm accuracy. Bill length was measured from the base of the culmen to the bill tip. Wing length was measured as the unflattened wing chord, from the carpal joint to the tip of the longest primary feather; before flight feathers emerged, we measured the distance from the carpal joint to the wing tip with the wing folded in the same posture. Tarsus length was measured with calipers reading with the jaws held perpendicular to the tarsometatarsus, spanning the entire tarsometatarsal segment. Tail length was defined as the distance from the point where the two central tail feathers emerge from the skin to the tip of the longest rectrix; when tail feathers were not fully developed, the length of the feather sheath was measured using the same posture.

All measurements were performed by a single researcher to minimize observer effects. Measurements taken on the last day before nestlings departed from the nest were considered as fledging size. We defined the nestling period as the time from the day the first egg hatched to the day the last nestling normally left the nest. The probability of producing replacement clutches is low for both species, owing to the brief breeding season in the alpine environment. Hence, despite not banding the parents, our data were unlikely to involve repeated monitoring of nests attended by the same adults. We made no attempt to distinguish the sex of nestlings because of their identical plumage during the early developmental stage. However, this did not affect our results because both rosefinch species are monomorphic in adult body mass.

### 2.3. Data Analysis

Our analyses focused on nestling mass because this trait is most sensitive to environmental conditions and is highly correlated with other body dimensions [20]. We fitted a logistic model, which has been demonstrated to be highly suitable for passerines [21], to describe the growth in body mass of each nestling. The equation is: body mass at age *t* = asymptotic body mass/[1 + exp(*b* + *kt*)], where *b* is a constant and *k* is the growth rate constant. Not all nestlings we found were measured through their complete growth period because they were found later, died, or due to a tight field schedule. Only individuals that were measured at least four times over the entire nestling period were used to estimate the growth parameters. Data included in the analysis consisted of 62 *C. rubicilloides* nestlings from 22 broods and 51 *C. eos* nestlings from 21 broods. On average, each nestling was measured 7.8 times (±2.8, range 4–15, *n* = 62) in the former species and 8.5 times (±2.6, range 6–15, *n* = 51) in the latter species, with no interspecific difference (Student’s *t*-test, *t* = 1.28, *df* = 111, *p* = 0.21). Therefore, we did not consider the potential effect of observation frequency on our comparison of growth.

Interspecific differences in nestling period length as the response variable were compared using a general linear model, with species the fixed factor, and nesting altitude, clutch start date, brood size and year as the covariates. For the interspecific comparison of growth rate as the response variable, because most broods contained more than one nestling and growth of siblings was not independent, we fitted a linear mixed model with nest identity as a random factor, using the same variables as in the nestling period analysis as fixed factors. Only the main effects of each explanatory variable were considered because the biological implications of interactions between these explanatory variables seemed less clear. We fitted the models using a Gaussian error distribution with an identity link function. Variance components were estimated using restricted maximum likelihood (REML), and degrees of freedom were approximated using the Satterthwaite method. Prior to these multivariate analyses, continuous data were converted to natural logarithms to improve normality.

We also compared the growth parameters of the two rosefinches with passerine values predicted from their body mass, and further contrasted them with two additional lowland congeners (the scarlet rosefinch *C. erythrinus* in Poland [22]; the house finch *C. mexicanus* in North America [23]). Predicted values of the growth rate constant *k* and nestling period were derived from the allometric equations established by Remeš and Martin [17] based on 115 passerine species (growth rate constant *k* = 0.72 W^−0.13^, nestling period = 0.77 W^0.15^). We calculated the observed and predicted time required to grow from 10% to 90% of the asymptotic body mass (*t*_10–90_) using the formula 4.39/*k*. Relative fledging mass (the percentage of adult body mass) was compared directly with the mean of the 115 passerine species, as the effect of body mass is already accounted for in this parameter.

All statistical tests were carried out using SPSS for Windows (Version 16.0; SPSS Inc., Chicago, IL, USA). Significance was assessed using two-tailed tests, and means are presented ± SD unless specified otherwise.

## 3. Results

### 3.1. Interspecific Comparison of Nestling Developmental Patterns Between the Two Alpine Rosefinch Species

The nestling periods averaged 14.0 days (±0.9, range 12–15, *n* = 18 broods) in *C. rubicilloides* and 13.5 days (±1.1, range 12–16, *n* = 26 broods) in *C. eos*. No statistical difference in this parameter was evident between the two species, as shown by a general linear model (*F*_1, 37_ = 1.67, *p* = 0.20) when accounting for nesting altitude, clutch start date, brood size, and year (all *p* > 0.28).

Development of nestlings of the two species was comparable for several morphological traits. Hatchlings were naked except for fine gray down on capital and scapulohumeral tracts. Eyelids remained closed for the first 3 days after hatching and opened fully between days 5 and 6. Females ceased brooding the nestlings by day 8, by which time feathers were well developed. However, there were interspecific differences in other traits. In *C. rubicilloides*, the calamus emerged from the ventral and spinal tracts on day 4, the primaries emerged from feather sheaths on day 6 or 7, and tail feathers on day 9. For *C. eos*, the corresponding events showed a delay of 1–2 days.

Interspecific difference in body mass at hatching was 62.5%, and the difference increased to 94.2% shortly before fledging (Table 1). These interspecific differences increased with nestling age and were also evident in linear measurements, and the magnitudes of the differences for ready-to-fledge nestlings were close to those observed between adults of the two species (Table 1). Despite their different ontogenetic patterns, nestlings of both species were similar in the proportion of each phenotypic trait relative to that of their female parents (*C. rubicilloides*: body mass 76.0%, bill 59.7%, wing 52.4%, tarsus 97.3%, tail 29.7%; *C. eos*: 77.0%, 66.3%, 54.6%, 95.5%, 26.5% in the same order).

### 3.2. Interspecific Comparison of Growth Parameters Between the Two Alpine Rosefinch Species

**Figure 1:**
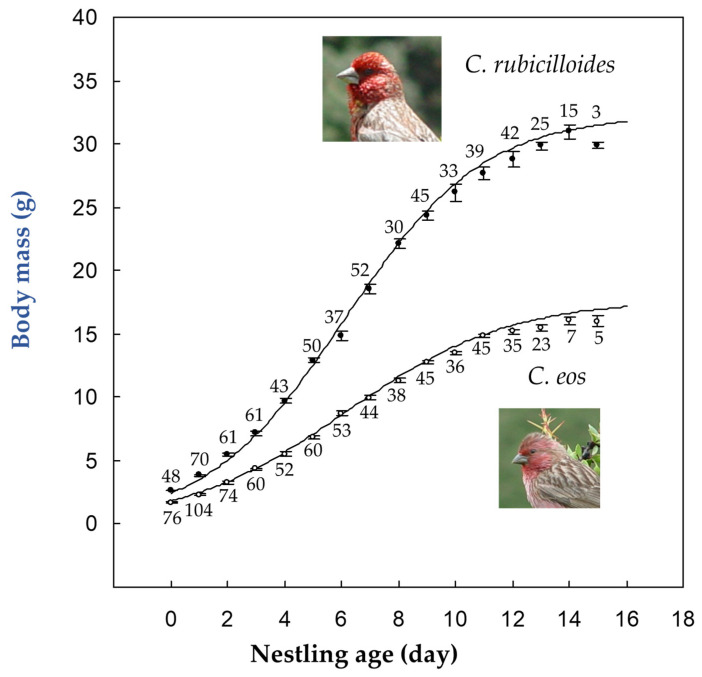
Growth trajectories of nestling body mass in the two alpine rosefinch species. Values are presented as mean ± SE. Because not all clutches were monitored from hatching to fledging, sample size differed at different age. The curves are derived from logistic models fitted to describe individual growth.

The growth rate constant *k* of the logistic equation for 62 *C. rubicilloides* nestlings averaged 0.41 g/day (±0.06), and was significantly larger than 0.35 g/day (±0.06) for 51 *C. eos* nestlings according to the linear mixed model analysis, which statistically accounted for nesting altitude, clutch start date, brood size, and year (Table 2). In *C. rubicilloides* nestlings, asymptotic body mass was 32.3 g (±4.0) and *t*_10–90_ was 11.4 days (±2.2). The corresponding values for 51 *C. eos* nestlings were 17.7 g (±2.4) and 12.5 days (±2.1) (Figure 1).

### 3.3. Interspecific Comparison of Growth Parameters Between the Two Alpine and the Two Lowland Rosefinch Species

Grouping species by altitude revealed a consistent direction of deviation from predicted values in the two lowland rosefinch species, while the two alpine rosefinch species deviated in the opposite direction for the same metrics (Table 3). Specifically, the two lowland rosefinches had growth rate constant *k* values that were higher than those predicted by the allometric equations fitted to 115 passerine species, whereas the two alpine rosefinches had growth rate constant *k* values lower than predicted. Consistently, *t*_10–90_ was lower than predicted in the lowland species but higher than predicted in the alpine species. By comparison, nestling period did not show a consistent direction of deviation in the two lowland species, while both alpine species had nestling period values longer than predicted. For relative fledging mass, the lowland species values were higher than predicted, whereas the alpine species values were lower than predicted.

## 4. Discussion

Across bird species, a tendency for larger species to grow slower and develop longer than smaller species is evident [17,24,25,26,27], most likely due to a basic design constraint [28]. However, the opposite was true for the growth trajectories of the two size-distinct rosefinch species. *C. rubicilloides* nestlings were 63% heavier than *C. eos* nestlings at hatching and up to 94% heavier when departing from the nest at the same age. Therefore, it was the significantly faster growth rate of *C. rubicilloides* nestlings that contributed to the 31% difference in mass gain between the two congeners. This increased interspecific divergence throughout the nestling period occurred similarly in several linear dimensions. The faster growth of *C. rubicilloides* nestlings also led to an earlier feather development compared to *C. eos* nestlings. Among three determinants of fledgling size (size at the starting point of growth, growth rate and length of time spent in growing), growth rate is the major contributor to interspecific differences in ontogeny. The result is consistent with reports for several *Geospiza* finches [29,30], but differs from those for two subspecies of *Passerella* sparrow [31] and two *Parus* tit species [32] where size differences at hatching are primarily responsible for the final size differences.

So what were the drivers for *C. rubicilloides* to adopt a faster growth strategy? A plausible driver was the availability of food resources required to raise nestlings. It is known that high growth rates often carry fitness costs, typically including starvation due to increased metabolic demands (reviewed in [7]). All other things being equal, fast growth is possible only when nestlings receive sufficient food from their parents [33,34]. Both rosefinch species are similar in general nesting habitats, breeding parameters (annual breeding attempts, clutch size, brood size and incubation period), nest predation rate and reproductive success [2,13]. This similarity allows our between-species comparison of growth parameters to exclude the potential effect of several growth-related factors such as ambient temperature [35], sibling competition and predation [26,36]. However, the two congeners differ in food availability, with *C. rubicilloides* nestlings, but not *C. eos* ones, potentially experiencing little food limitation [2]. Moreover, the greater energy requirements of *C. eos* nestlings may result from the high costs of thermoregulation associated with their small body size.

In the alpine zones, the leguminous seeds on which *C. rubicilloides* parents feed their young ripen synchronously over a narrow time window (between early August and early September); in contrast, the diet of *C. eos* nestlings, consisting of a variety of grass seeds, is available for an extended period (from mid-August up to early October). In response to this species-specific temporal pattern of food availability, *C. rubicilloides* lay eggs over approximately 30 days (between early July and early August), while *C. eos* do so for around 50 days (from mid-July to early September) [2,13]. Moreover, *C. rubicilloides* prefer nesting in taller bushes but *C. eos* tend to place their nests in lower, densely branched bushes, although the nest plant species selected do not differ between them [2]. *C. rubicilloides* nests become increasingly exposed to potential predators and inclement weather due to seasonal degradation of the foliage that provides shelters for the nests; this problem appears less serious for *C. eos* because the low, dense bushes may provide better concealment for their nests. Hence, the rapid growth of *C. rubicilloides* nestlings to minimize the period of vulnerability should be adaptive in the face of seasonal time constraints. A similar growth strategy has been observed in several other bird species [25,26,37,38]. In addition, based on our preliminary comparisons, although nestlings of *C. rubicilloides* appeared to adopt an adaptive strategy of relatively rapid growth, multiple growth parameters still indicate that, relative to closely related lowland species, their development remains strongly constrained by the high-elevation environment. This finding is consistent with the conclusion of a previous study [27].

Growth, as a critical stage of the avian life cycle, is subject to natural selection. Our study sheds light on the adaptive radiation of *Carpodacus* rosefinches in high-altitude Asia [39,40]. Sympatric speciation can occur through phenotypic divergence driven by ecological diversification to avoid competition for shared resources [41], as demonstrated in Darwin’s finches in the Galápagos Archipelago [42]. It appears to be the case for *Carpodacus* rosefinches in alpine habitats, where competition for food resources is intense [2]. The present data reveal that the difference in adult size may be attributed to an evolutionary shift in the ontogenetic trajectory arising from trophic niche segregation between the two congeners.

## 5. Conclusions

Overall, our results show that ecological constraints can override the typical size-related slowing of growth in passerines. In alpine zones above 4300 m in south Tibet, the larger streaked rosefinch *C*. *rubicilloides* displayed a higher nestling mass growth rate than the smaller pink-rumped rosefinch *C*. *eos*, with *k* values of 0.41 g/day and 0.35 g/day, respectively, while nestling periods were similar at 14.0 and 13.5 days. Growth curves supported a significant species difference in growth rate. These patterns align with strong trophic niche segregation, as *C. rubicilloides* relies mainly on abundant, productive legume seeds, whereas *C. eos* uses smaller grass seeds that likely impose higher searching costs. We suggest that reliable food supply and limited seasonal time can overcome the allometric constraints imposed by body size, which favors rapid growth and thus reduces exposure to harsh weather and other risks.

## Figures and Tables

**Table 1:** Body mass and linear dimensions of nestlings at hatching and near fledging (≤1 day) of the two alpine rosefinch species, along with interspecific differences (%) in these traits and the percentage of interspecific in adult female body size (%, marked with *). Sample size is provided in parentheses.

	Hatchlings			Fledglings			
Body Traits	*C. rubicilloides*	*C. eos*		*C. rubicilloides*	*C. eos*		
	Mean ± SD	Mean ± SD	%	Mean ± SD	Mean ± SD	%	% *
Body mass (g)	2.6 ± 0.3 (29)	1.6 ± 1.8 (55)	62.5	30.1 ± 1.8 (55)	15.5 ± 1.2 (40)	94.2	97.0
Bill length (mm)	3.0 ± 0.4(29)	2.6 ± 0.1 (55)	16.6	7.9 ± 0.9 (57)	5.9 ± 0.3 (34)	33.9	49.4
Wing length (mm)	6.9 ± 0.4 (18)	5.8 ± 0.5 (55)	18.6	52.9 ± 5.7 (56)	40.0 ± 5.0 (36)	32.3	38.0
Tarsus length	6.4 ± 0.5 (29)	5.2 ± 0.4 (55)	23.2	22.3 ± 0.8 (56)	17.0 ± 0.8 (38)	31.2	28.7
Tail length (mm)	−	−	−	25.3 ± 4.1 (55)	20.0 ± 3.7 (35)	26.5	27.9

**Table 2:** Results of a general linear mixed model analysis testing the effects of potential explanatory variables on the average growth rate of body mass of nestlings within broods of the two alpine rosefinch species (62 nestlings from 22 *C. rubicilloides* broods; 51 nestlings from 21 *C. eos* broods), with nest identity included as a random term (*z* = 3.52, *p* < 0.001).

Explanatory Variables	*Num df*	*Den df*	*F*	*p*
Intercept	1	36.3	1.18	0.28
Species	1	36.7	8.12	0.01
Nesting altitude	1	36.3	1.32	0.26
Clutch start date	1	41.2	0.03	0.86
Brood size	1	38.6	0.08	0.77
Year	2	35.9	1.33	0.28

**Table 3:** Observed and predicted growth parameters of the two alpine rosefinches and two lowland congeners. Predicted values were derived from allometric equations from [17].

Growth Parameters	*C. rubicilloides* (~40 g)		*C. eos* (~20 g)		*C. erythrinus* (~23 g)		*C. mexicanus* (~20 g)	
	Obs.	Pre.	Obs.	Pre.	Obs.	Pre.	Obs.	Pre.
Growth rate constant *k* (g/day)	0.41	0.45	0.35	0.49	0.55	0.48	0.63	0.49
*t*_10–90_ (day)	11.7	9.8	12.5	9.0	8.0	9.1	7.0	9.0
Nestling period (day)	14.0	13.5	13.5	12.2	11.7	12.4	15.0	12.2
Relative fledging mass (%)	76	82	77	82	83	82	85	82

## Data Availability

Further information on the data included in this study is available from the corresponding author upon reasonable request.

## References

[1] Clement P., del Hoyo J., Elliott A., Sargatal J., Christie D.A., de Juana E. (2020). Streaked Rosefinch (*Carpodacus rubicilloides*), version 1.0. Birds of the World.

[2] Lu X., Gong G.H., Ma X.Y. (2011). Niche segregation between two alpine rosefinches: To coexist in extreme environments. Evol. Biol..

[3] Killpack T.L., Karasov W.H. (2012). Growth and development of house sparrows (*Passer domesticus*) in response to chronic food restriction throughout the nestling period. J. Exp. Biol..

[4] Remeš V., Matysioková B., Vrána J. (2020). Adaptation and constraint shape the evolution of growth patterns in passerine birds across the globe. Front. Zool..

[5] Sauve D., Friesen V.L., Charmantier A. (2021). The effects of weather on avian growth and implications for adaptation to climate change. Front. Ecol. Evol..

[6] Kapali G.P., Callier V., Gascoigne S.J., Harrison J.F., Shingleton A.W. (2022). The steroid hormone ecdysone regulates growth rate in response to oxygen availability. Sci. Rep..

[7] Dmitriew C.M. (2011). The evolution of growth trajectories: What limits growth rate?. Biol. Rev..

[8] Potapov R.L. (2004). Adaptation of birds to life in high mountains in Eurasia. Acta Zool. Sin..

[9] Lu X. (2005). Reproductive ecology of Blackbirds (*Turdus merula maximus*) in a high-altitude location, Tibet. J. Ornithol..

[10] Lu X. (2006). Abundance and breeding ecology of Brown Accentors *Prunella fulvescens* in Lhasa, Tibet. Acta Ornithol..

[11] Lu X. (2008). Breeding ecology of an Old World high-altitude warbler, *Phylloscopus affinis*. J. Ornithol..

[12] Lu X., Gong G.H., Ma X.Y., Ke D.H. (2009). Breeding biology of the White-browed Tit-warbler (*Leptopoecile sophiae*) in alpine shrubs, southern Tibet. Condor.

[13] Gong G.H. (2005). Breeding Ecology of Two *Carpodacus rosefinch* Species (*C*. *rubicilloides* and *C. eos*) in Alpine Zones Around the Mid-Yalong Zangbo River, Tibet. Master’s Thesis.

[14] Herrel A., Podos J., Huber S.K., Hendry A.P. (2005). Bite performance and morphology in a population of Darwin’s finches: Implications for the evolution of beak shape. Funct. Ecol..

[15] Van der Meij M.A.A., Bout R.G. (2006). Seed husking time and maximal bite force in finches. J. Exp. Biol..

[16] Gebhart-Henrich S.G., Richner H., Starck J.M., Ricklefs R.E. (1998). Causes of growth variation and its consequences for fitness. Avian Growth and Development.

[17] Remeš V., Martin T.E. (2002). Environmental influences on the evolution of growth and developmental rates in passerines. Evolution.

[18] Eckerström-Liedholm S., Sowersby W., Gonzalez-Voyer A., Rogell B. (2017). Time-limited environments affect the evolution of egg-body size allometry. Evolution.

[19] Lu X., Zhang L.Y., Zeng X.H. (2007). Comparisons of the alpine bird communities across habitats and between autumn and winter in the mid-Yalong Zangbo River valley, Tibet. J. Nat. Hist..

[20] O’Connor R.J., Peaker M. (1975). Growth and metabolism in nesting passerines. Advances in Avian Physiology.

[21] Ricklefs R.E. (1968). Patterns of growth in birds. Ibis.

[22] Stjernberg T. (1979). Breeding biology and population dynamics of the Scarlet Rosefinch *Carpodacus erythrinus*. Acta Zoologica Fennica.

[23] Badyaev A.V., Martin T.E. (2000). Individual variation in growth trajectories: Phenotypic and genetic correlations in ontogeny of the House Finch (*Carpodacus mexicanus*). J. Evol. Biol..

[24] Ricklefs R.E., Starck J.M., Starck J.M., Ricklefs R.E. (1998). Embryonic growth and development. Avian Growth and Development.

[25] Tjørve K.M.C., García-Peña G.E., Székely T. (2009). Chick growth rates in Charadriiformes: Comparative analyses of breeding climate, development mode and parental care. J. Avian Biol..

[26] Martin T.E., Lloyd P., Bosque C., Barton D.C., Biancucci A.L., Cheng Y.R., Ton R. (2011). Growth rate variation among passerine species in tropical and temperate sites: An antagonistic interaction between parental food provisioning and nest predation risk. Evolution.

[27] Guo Y.Y., Gao H.X., Lu X. (2025). High-elevation birds grow more slowly but to heavier weights than low-elevation birds. Oecologia.

[28] West G.B., Brown J.H., Enquist B.J. (2001). A general model for ontogenetic growth. Nature.

[29] Grant P.R. (1981). Patterns of growth in Darwin’s finches. Proc. R. Soc. B.

[30] Boag P.T. (1984). Growth and allometry of external morphology in Darwin’s finches (*Geospiza*) on Isla Daphne Major, Galapagos. J. Zool. Lond..

[31] Burns K.J. (1993). Geographic variation in ontogeny of the fox sparrow. Condor.

[32] Björklund M. (1996). Similarity of growth among great tits (*Parus major*) and blue tits (*P*. *caeruleus*). Biol. J. Linn. Soc..

[33] Martin T.E. (1987). Food as a limit on breeding birds: A life-history perspective. Ann. Rev. Ecol. Syst..

[34] Schew W.A., Ricklefs R.E., Starck J.M., Ricklefs R.E. (1998). Developmental plasticity. Avian Growth and Development.

[35] Podlesak D.W., Blem C.R. (2001). Factors associated with growth of nestling prothonotary warblers. Wilson Bull..

[36] Nilsson J.Å., Gårdmark A. (2001). Sibling competition affects individual growth strategies in marsh tit, *Parus palustris*, nestlings. Anim. Behav..

[37] Ricklefs R.E. (1976). Growth rates of birds in the humid New World tropics. Ibis.

[38] Cox W.A., Martin T.E. (2009). Breeding biology of the three-Striped warbler in Venezuela: A contrast between tropical and temperate Parulids. Wilson J. Ornithol..

[39] Landmann A., Winding N. (1995). Adaptive radiation and resource partitioning in Himalayan high-altitude finches. Zoology.

[40] Landmann A., Winding N. (1995). Guild organisation and morphology of high-altitude granivorous and insectivorous birds: Convergent evolution in an extreme environment. Oikos.

[41] Bolnick D.I., Fitzpatrick B. (2007). Sympatric speciation: Theory and empirical data. Ann. Rev. Ecol. Evol. Syst..

[42] Tebbich S., Sterelny K., Teschke I. (2010). The tale of the finch: Adaptive radiation and behavioural flexibility. Phil. Trans. R Soc. B.

